# Temperature Dependence of Space Charge Retention in Bulk Acrylic

**DOI:** 10.1002/advs.78400

**Published:** 2026-10-31

**Authors:** Bryson C. Clifford, Kathryn M. Sturge, Noah Hoppis, Emily H. Frashure, John Cumings, Timothy W. Koeth

**Affiliations:** ^1^ Department of Materials Science and Engineering University of Maryland USA

**Keywords:** dielectrics, electrical breakdown, electrical treeing, space charge, thermally activated processes

## Abstract

Charge loaded dielectrics (CLDs) are created when a dielectric is exposed to high‐energy charged particle radiation and accumulates an internal space charge. The material behavior of CLDs is not as well understood as that of non‐irradiated dielectrics. Specifically, two characteristic processes remain under investigated: (*i*) the slow, quasistatic charge dissipation (minutes to hours timescale) and (*ii*) the spontaneous dielectric breakdown event (sub‐microsecond timescale). Herein the temperature dependence of space charge retention in electron‐irradiated bulk acrylic is probed by thermally manipulating charge carrier mobility over a temperature range of –80°C ≤ *T* ≤ 0°C. Charge carrier mobility in bulk acrylic, proportional to charge dissipation, was able to be restricted indefinitely when bulk samples were stored below a critical temperature found to be in the range of –50°C ≤ *T* ≤ –40°C. Above this critical temperature, the amount of transferred charge measured during each breakdown decreased exponentially. A similar temperature‐dependence in the electrical treeing pattern morphology is observed post dielectric breakdown as direct physical evidence of the plasma created during breakdown. These observed temperature‐dependencies suggest space‐charge mobility in electron‐irradiated bulk acrylic is dominantly phonon‐dependent and strongly influenced by the β‐relaxation temperature of acrylic, which falls within the –50°C ≤ *T* ≤ –40°C critical temperature range.

## Graphical abstract

The temperature dependence of space charge stability in electron‐charged bulk acrylic is probed by thermal manipulation. Charge carrier mobility is observed to be phonon‐dependent, which can be restricted indefinitely below a critical temperature. Electrical tree morphology due to spontaneous dielectric breakdown also varies with temperature, suggesting the temporary localization or delocalization of shallow trapping sites significantly influences charge loaded dielectric behavior.

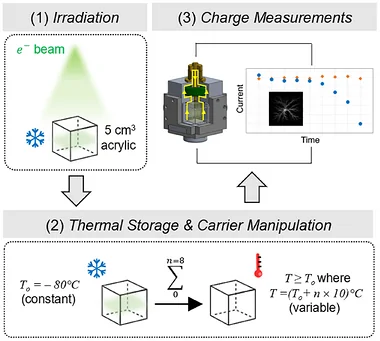

## 1 Introduction

Dielectric deterioration and breakdown are of concern in many electronic and electrical applications such as medium‐ and high‐voltage components, electrical transformers, and gas‐insulated switches [1, 2, 3, 4]. In most laboratory and field settings, dielectric breakdown occurs when an applied *external* electric field exceeds the material‐specific polarizability threshold of the dielectric. It is understood that repeat partial discharge events (e.g., corona rings, micro‐sparking) are caused by electric field enhancements around geometric features (e.g., small bend radius, triple junction points) and often localized to areas of material flaws (e.g., low‐density or cavity regions), which eventually lead to the full breakdown of the dielectric (e.g., sustained electrical arc) [5, 6, 7, 8, 9].

A charge loaded dielectric (CLD) is any dielectric material that has accumulated an excess of *internal* electric space charge from high‐energy charged particles. This can be a result of extreme environments (i.e., radiation) or as a result of the induced buildup of surface charge from AC or DC current, injecting charge into the bulk. Dielectric breakdown of a CLD with internally accumulated space charge thus occurs when the space‐charge‐induced *internal* electric field exceeds the material‐specific polarizability threshold of the dielectric. The effects of space charge add complexity on top of traditional dielectric breakdown understanding, some examples of which include anomalous spacecraft charging and failure, dielectric window failure, and in predicting CLD lifetime before breakdown [10, 11, 12, 13]. CLD phenomena i.e., space charge accumulation, retention, and quasistatic dissipation are generally known [14, 15, 16], but until recently empirical observations directly correlating space charge with the breakdown process or resulting electrical treeing has been lacking.

The “spontaneous breakdown” language used in this work refers to the extremely fast < 1 µs, catastrophic dielectric breakdown event (DBE), which is distinct from but related to the CLD phenomena mentioned above. A DBE occurs when the local electric field at a defect site exceeds the dielectric threshold strength of the material. From this point, a propagating electrical breakdown front is formed via electron avalanche, creating a highly conductive network of plasma channels; allowing the system to reconfigure charge and achieve a lower, more energetically stable state. Spontaneous charge reconfiguration appears highly stochastic and results in permanent damage‐artifacts that span the surface or interior bulk. Commonly referred to as electrical trees or Lichtenberg figures (LFs) in literature, these distinctively branched patterns often contain one large main channel that connects the breakdown initiation point to the volume or area with highest accumulated space charge density. Smaller channels stem from the main channel in a fractal‐like nature [17, 18, 19]. During the DBE, light, sound, and mechanical disturbances are produced as the plasma channel network grows and ceases. Each disturbance has disparate characteristic times scales, making the design of in situ laboratory experiments with sufficient sensitivity, resolution, and acquisition speed a significant challenge.

Recent works have directly probed space charge phenomena by implementing novel experimental setups for CLD analysis in a laboratory setting. Hoppis et al. designed a novel optical image delay line enabling in situ high‐speed (GHz frame rate) optical imaging of space‐charge‐induced DBEs in bulk acrylic with high success rate [20]. This unconventional setup enabled the collection of empirical data with which (*i*) Sturge et al. reported the observation of two different breakdown propagation modes i.e., branch‐type and ivy‐type, measured to propagate at speeds exceeding 10^6^ and 10^7^ m s^−1^, respectively [21, 22], and (*ii*) Montano et al. used to create and inform a hybrid resistor‐inductor‐capacitor (RLC) circuit model with a grid‐based network to computationally predict realistic electrical tree formation in space‐charged bulk acrylic [23]. It is presently a matter of debate whether the branch‐type and ivy‐type breakdown modes are different physical mechanisms or rather overlapping, simultaneously plausible processes governed by preferred relative free energy pathways available at the time of spontaneous dielectric breakdown initiation. Regardless and remarkably, both types of electromechanical damage propagate faster than the mechanical speed of sound in acrylic (reported as 2.74 × 10^3^ m s^−1^ at 22°C, for acrylic with 120 kg mol^−1^ molecular weight) [24].

Other novel experimental setups are important to note. For example, a pulsed electroacoustic (PEA) setup was fabricated to measure the space charge distribution in bulk acrylic (15.4 × 15.4 cm cuboid, 2.41 cm thick) immediately after 6 MeV electron beam irradiation at room temperature [25]. PEA measurements found both positive and negative charge density layers are created inside bulk acrylic by the incident electron deposition, which is the first report of a positive charge layer generated in acrylic due to electron irradiation in literature. A novel hardware setup using a 200 amu residual gas analyzer (RGA) was fabricated to detect gaseous by‐products produced immediately after the spontaneous breakdown of bulk acrylic (5 cm cubic) CLDs [26]. Initial RGA measurements suggest two major types of discharge products are produced by bond dissociation and/or Joule heating within the material bulk: (*i*) low intensity hydrocarbons, which peak then quickly decay and (*ii*) high intensity burn products (i.e., H_2_, CO, and CO_2_) which are either constant or increase in intensity over the measured period.

A custom‐built, low‐inductance test fixture for current waveform acquisition during space‐charge‐induced dielectric breakdown was previsouly designed and fabricated to accomodate bulk CLD samples [27]. Implementation of this hardware enabled the exploration of time‐dependent space charge retention as a function of incident electron beam current in room temperature electron irradiated bulk acrylic [28]. Acrylic samples irradiated with a 3 mA incident beam current were found to accumulate less space charge but retain space charge for a longer duration (∼45 min) compared to identical samples irradiated at 6 and 9 mA beam currents (with retention duration ∼30 min). Most recently, a laboratory‐scale streak camera‐based velocimeter setup was fabricated and used to directly measure breakdown velocity as a function of imbedded charge density and temperature in electron irradiated bulk acrylic samples, offering similar spatial and temporal resolution, improved 1D data collection, and reduced cost compared to Gated Image Intensifier‐based framing camera setups [29]. The streak camera‐based velocimetry setup was used to separately measure ivy‐type breakdown propagation speeds in electron irradiated bulk acrylic, verifying the novel technique and validating the 10^6^ m/s breakdown velocities reported by Sturge et al. [21].

Herein, we probe the thermal dependence of space charge retention in bulk poly(methyl methacrylate) (PMMA or acrylic) by thermally manipulating space charge mobility over the temperature range of –80°C ≤ *T* ≤ –0°C. CLDs were created by irradiating dielectric specimens, pre‐chilled to –80°C, with a 4.5 (+3% –2%) MeV electron Dynamitron at a current of 3 mA. As the energetic electrons bombard the specimen, electrons of various energies become embedded inside the bulk as spatially distributed excess charge (i.e., space charge). At pre‐determined temperatures, CLD samples were mechanically insulted by a sharp object to initiate a space‐charge‐induced DBE. No external electric field was applied at the time of mechanical insult. Using the custom‐built, low‐inductance test fixture for current waveform acquisition mentioned above, the electrical current reconfigured by each breakdown event was captured and integrated over time to quantify the amount of transferred charge per event, which was then plotted against bulk sample temperature at the time of induced breakdown. Corresponding optical images of each LF produced in control and experimental sample sets were obtained. Unique periodic damage‐artifacts are discussed, along with a brief discussion on the possible charge mobility mechanisms in PMMA, inferred from these temperature‐dependent observations.

## 2 Results and Discussion

All samples were pre‐cooled to –80°C before the implantation of excess electrons. Control samples were held at –80°C for 27 consecutive days post‐irradiation. Regardless of when breakdown was induced, the space‐charge‐loaded PMMA control samples transferred an average of 4.9 ± 0.2 µC of charge during dielectric breakdown, as shown in Figure 1 inset. The control samples exhibited < 1.89 standard error over 27 days and < 3% ensemble fluctuation from average, shown in Table S1 and Figure S1, respectively. Experimental samples that were equilibrated at intervals in the temperature range of –70°C ≤ *T* ≤ –50°C produced 6% less charge on average compared to the control sample average. However, when experimental samples were equilibrated at intervals in the temperature range of –40°C ≤ *T* ≤ 0°C, an exponential decrease in transferred charge was observed at each Δ*T* = +10°C increment i.e., 7%, 13%, 32%, 53%, and 100% less compared to the control sample average. These results are shown in Figure 1 with exponential fit (*R^2^* = 0.9961) shown in Figure S2.

**FIGURE 1:**
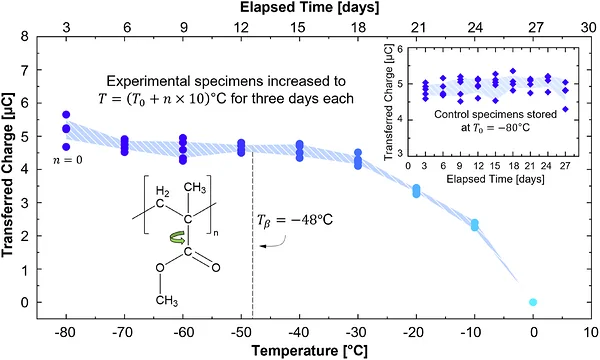
Temperature‐dependent space charge retention. Quantified amount of charge produced per space‐charge‐induced dielectric breakdown event (transferred charge, µC) in bulk transparent PMMA as a function of sample temperature at the time of induced breakdown (temperature, °C). The transferred charge of experimental samples (circles) significantly decreased when samples were equilibrated above the *β*‐transition temperature *T*_β_ of PMMA (dashed line), compared to control specimens (diamonds, inset), which exhibited minimal variation in transferred charge over the duration of the ∼30‐day experiment. The shaded, hatched area on both graphs corresponds to 95% confidence interval. The blue color of the data points lightens with increasing temperature.

Due to the qualitative nature of this work, several key assumptions have been made. Particularly, the amount of “transferred charge” is used synonymously with “retained charge”. However, equating these two quantities is an over‐simplification. During dielectric breakdown, some energy is lost due to plasma effects (i.e., ionization, Joule heating, UV‐radiation, carbon deposition, physical instabilities, etc.), mechanical deformation (i.e., elastic and plastic), and acoustic and optical effects. The amount of transferred charge during each DBE is thus expected to be less than the total amount of accumulated charge during irradiation and less than the total amount of retained charge, as some phonon effects are always present above absolute zero.

The plot of transferred charge vs temperature demonstrates two regimes separated by a critical temperature within the range of –50°C ≤ *T* ≤ –40°C. This critical temperature is in strong agreement with the β‐relaxation temperature (*T_β_*) of PMMA, reported as –48°C [30]. When *T* > *T_β_*, short‐range local conformational motion exists due to the molecular rotation of the ester side group (RCOOR’) about its bond to the main PMMA backbone. We hypothesize the temperature‐dependent movement of the ester pendant group directly facilitates charge carrier (i.e., electron or hole) mobility – resulting in an inverse relationship between CLD sample temperature and space charge retention. Our experimental current measurements are in agreement with the temperature‐dependent electric strength theory for linear polymers published by Ieda et al. [31]. However, it is important to note that PMMA is lightly branched in structure, not exclusively linear.

The type of low linear energy, electron radiation used in this experiment is known to degrade PMMA dominantly through chain scission [32]. Random segmentation of the ultra‐high molecular weight PMMA creates numerous polymer segments of various lengths and thus of various ionization or electron affinity energies. The full list of additives (e.g., plasticizers, UV‐stabilizers, etc.) in commercially obtained PMMA is not reported, thus, it is plausible that some amount of conjugated rings are present. The presence of both various chain lengths and conjugated rings likely creates a high degree of delocalized molecular orbital overlap, similar to that observed in highly conjugated polymers [33], enabling numerous temperature‐sensitive shallow trapping sites for charge carriers. The focus of this work is on connecting experimental observations, supported by quantitative current measurements, to potential charge carrier mechanisms. However, additional concepts regarding polymer properties (molecular weight, tacticity, free volume, chain entanglement, etc.) [34, 35, 36, 37] and electron beam interactions (end chain radicals, radiation‐induced conductivity) [38, 39, 40] need to be considered for a full understanding of PMMA CLD behavior.

The current measured at the time of breakdown is fundamentally related to the magnitude of the electric field inside the CLD just before breakdown. By extension, it is assumed the current measured at the time of breakdown is directly proportional to the amount of space charge density available inside the CLD. This assumption holds in accordance with our quantitative current measurements: more charge is transferred when CLD samples are discharged at colder temperatures. We hypothesize temperature itself is a proxy for space charge density. For example, at very cold temperatures, minimal space charge is lost due to internal phonon movement (i.e., negligible charge carrier mobility), available shallow trapping sides induced by irradiation are highly localized, and thus more space charge (i.e., higher space charge density) is locally available to participate in the DBE. Higher space charge density in a material with a widened chain segment length distribution due to electron beam irradiation likely also has enhanced electron‐phonon coupling, which is known to broaden the tail states in the density of states (DOS) distribution in organic semiconductors [41]. Acrylic CLD samples held at colder temperatures may have a broadened DOS localized or “frozen in place” providing energetically favorable access to sufficiently filled charge traps, which are available to aid trap‐assisted recombination during dielectric breakdown. The degree to which trap‐assisted recombination occurs, may explain the difference between ivy‐type and branch‐type breakdown propagation modes.

Stark visual differences were also observed in LF morphology, shown in Figure 2, when samples were discharged at colder (*T* = –80°C) vs. warmer (*T* = –10°C) temperatures. At colder temperatures, the resulting LFs contain both ivy‐type and branch‐type channels. While at warmer temperatures, the resulting LFs contain only branch‐type channels (no ivy‐type channels observed). Under the conditions of this experiment, the probability of breakdown via the ivy‐type breakdown propagation mode becomes significant when breakdown is induced at a bulk sample temperature within ± 10°C of *T_β_*. The type of LF morphology visible a posteriori, is direct evidence of an inverse relationship between bulk sample temperature and internal space charge density. Optical images of all LFs from the control and experimental sample sets are shown in Figures S3 and S4, respectively.

**FIGURE 2:**
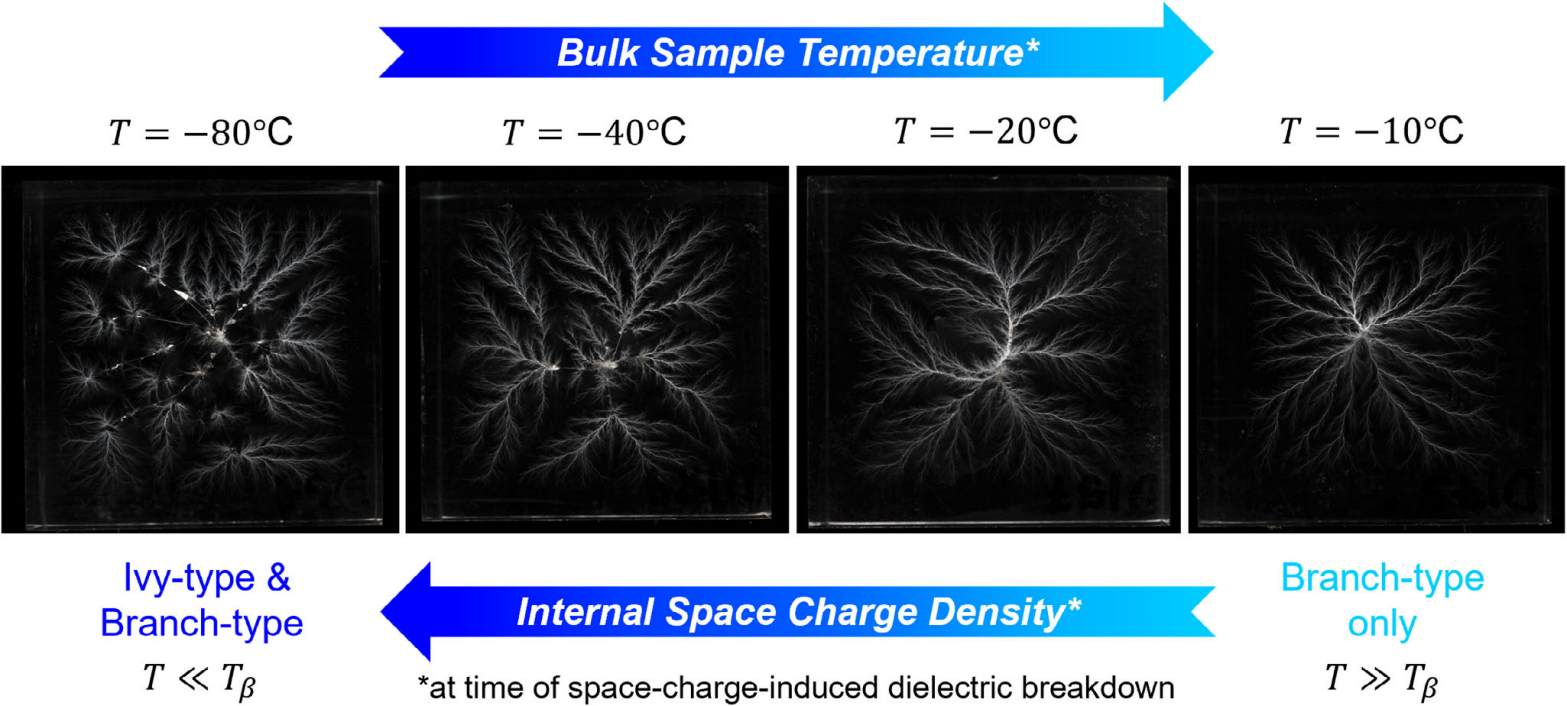
Bottom‐illuminated optical images of Lichtenberg figures (LFs) produced by space‐charge‐induced dielectric breakdown in transparent bulk PMMA at bulk sample temperatures of (left to right) −80°C, −40°C, −20°C, and 0°C. Samples were illuminated with a white LED from below in a dark room to enhance contrast between the void pathways (white) and the transparent bulk material (black). LFs containing both ivy‐type and branch‐type (left‐most) vs. only branch‐type (right‐most) channels are consistently produced when bulk sample temperature is *T* ≪ *T_β_* and *T* ≫ *T_β_*, respectively, at the time of space‐charge‐induced dielectric breakdown. Lighter blue colors (arrows and text) correspond to warmer temperatures, darker colors correspond to colder temperatures.

LFs containing (*i*) ivy‐type and branch‐type channels, and (*ii*) branch‐type channels only, are enlarged and shown for comparison in Figure 3a,b, respectively. Macroscopically, ivy‐type channels appear as thin lines in the LF, which transition into larger diameter, branch‐type channels at nodes of mechanical damage. Microscopically, these thin lines undulate in a periodic nature with an average channel radius on the order of ∼10 µm and an approximate wavelength between 25 < λ < 100 µm. No periodicity is observed in branch‐type channels. Polarized light contrast reveals ivy‐type channels are accompanied by a distorted periodic boundary layer surrounding the channel in the radial direction, with an inner radius equivalent to the channel radius, and an outer radius of *r_b_* ∼ 30 µm (Figure 3c). Contrast due to polarized light suggests this distortion is a physical density change in the bulk acrylic material, likely as the material surrounding the channel momentarily reached its glass transition temperature due to Joule heating or UV‐radiation by the plasma formed during breakdown. The boundary layer crests exhibit a slight phase‐shift compared to the channel periodicity. Other ivy‐type sections clearly show each crest of the periodic channel is fed by smaller streamers that are consistently off‐center, tending toward the direction of escaping gas (i.e., toward the DBE initiation point) (Figure 3d).

**FIGURE 3:**
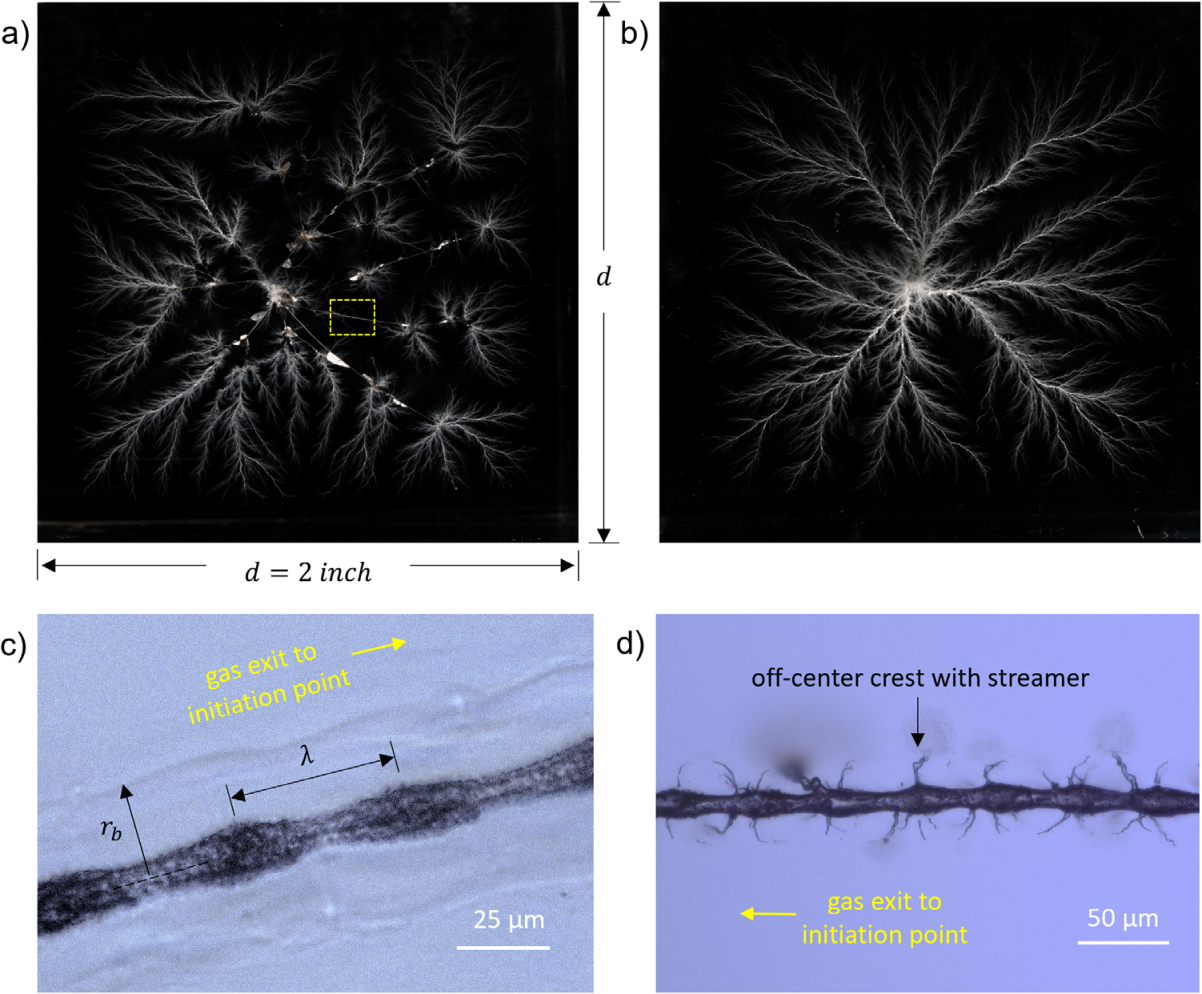
Bottom‐illuminated optical image comparison of (a) ivy‐type and (b) branch‐type characteristic LF from induced dielectric breakdown in 5 cm cubic samples of bulk PMMA. The transparent bulk sample appears black, the channel void space appears white, the yellow dashed box encloses a thin channel, representative of those which typically contain the periodic structures unique to ivy‐type LFs. No thin lines are observed in image (b). Polarized light contrast, digital microscope images of different periodic sections exhibiting (c) a PMMA density change in the boundary layer, with radius denoted *r_b_*, surrounding the channel, and (d) multiple off‐center peak amplitude crests connected to streamers. The peak crest position generally leans toward the direction of the dielectric breakdown initiation point.

For comparison, optical images of an (*i*) ivy‐type and (*ii*) branch‐type channel were taken after samples were cleaved longitudinally along the channel length, such that the channel void space is exposed at the surface (Figure 4a,b, respectively). The resulting surface roughness of these fractured samples can have an average topography exceeding ± 1 mm (Figure 4c), making direct characterization inside the channels a challenge. Nonetheless, Schwartz et al. used direct optical observations, similar to those reported here, and Raman mapping of a fractured periodic ivy‐type section to evaluate three theoretical plasma growth instability models [42]. Schwartz concluded that during the plasma discharge phase of the spontaneous breakdown process, the non‐adiabatic temperature gradient of the dense plasma channel drives a z‐pinch entropy mode, resulting in a physical instability that simultaneously couples and misaligns plasma density and temperature gradients while maintaining constant pressure, resulting in the periodic channels observed in ivy‐type LFs. The work of Schwartz et al. is important as it connects experimental observations with fundamental plasma theory and represents the first description of a plasma instability creating permanent features in a solid material. The optical images of periodic structures provided here were chosen to expand upon those already published by Schwartz et al.

**FIGURE 4:**
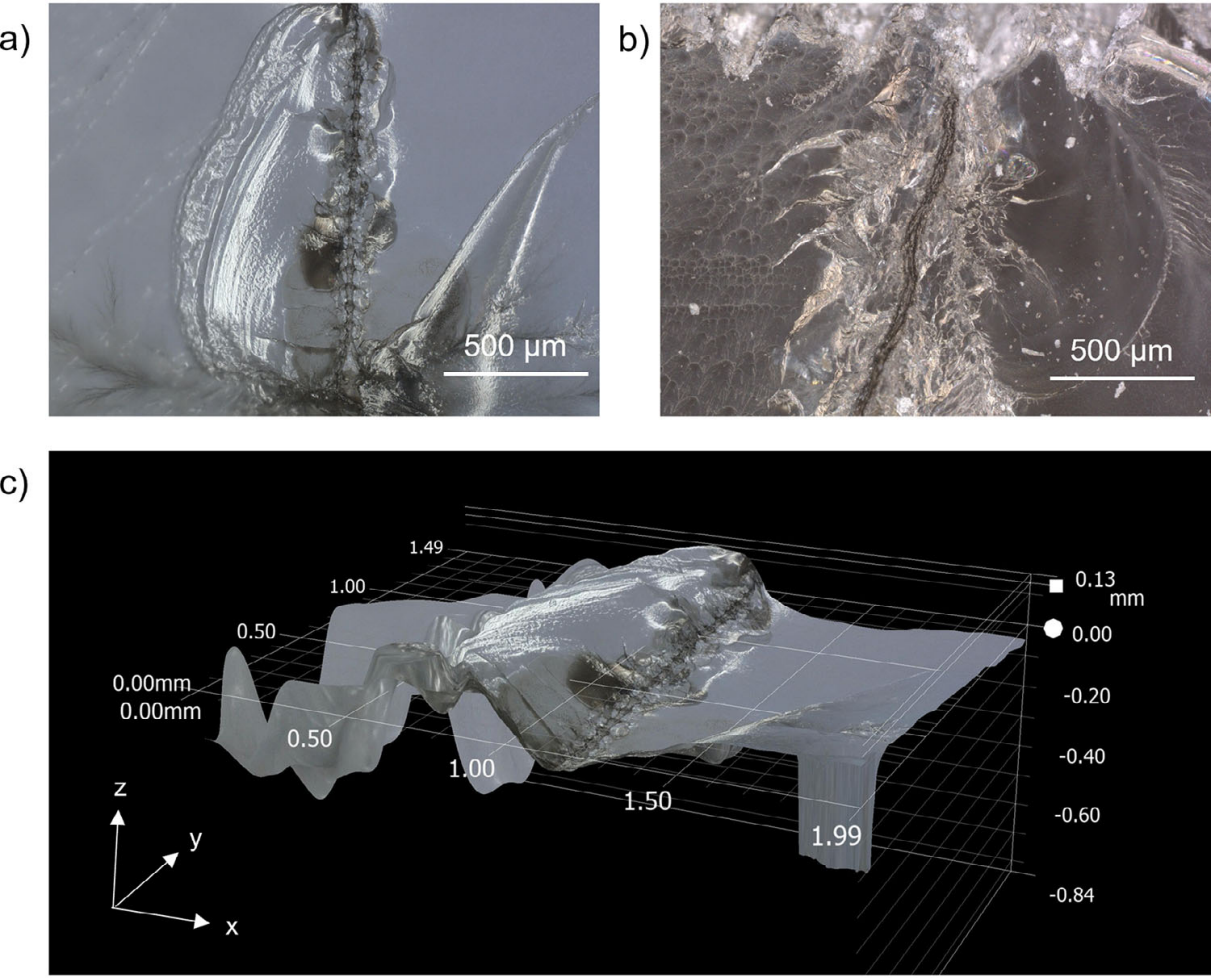
White‐light z‐stack digital optical images of LF damage in transparent bulk PMMA after bulk samples were fractured open, exposing (a) a periodic section of an ivy‐type channel and (b) a section of a branch‐type channel near the mechanical initiation point. (c) Corresponding digital topography map of the area in image (a), depicting typical mm‐scale roughness of the fractured surface.

## 3 Conclusion

Improved understanding of CLD behavior has both fundamental and applied merits in extreme environment applications such as electronics in space, radiation shielding, and high‐voltage technology. Novel experimental setups capable of direct measurements of the spontaneous breakdown of dielectrics or which probe CLD (i.e., space charge) phenomena are necessary for acquiring new insights. This work utilized direct optical observations and quantitative current measurements during breakdown to explore fundamental CLD phenomena in electron irradiated bulk PMMA. Samples were irradiated and stored at –80°C, then induced to breakdown at temperature increments of Δ*T* = +10°C, over a temperature range of –80°C ≤ *T* ≤ 0°C and 27 days. Current measurements were obtained from each breakdown, after which optical images of the corresponding LFs were acquired for comparison – establishing a correlation between temperature, transferred charge during breakdown, and resulting LF morphology.

The two breakdown propagation modes known to occur in space‐charge‐loaded PMMA (ivy‐type vs. branch‐type) were observed to produce different channel morphologies (periodic vs. non‐periodic, respectively), and measurably different amounts of transferred charge during breakdown when temperature is used as the dependent variable. The probability of finding periodic ivy‐type channels in the resulting LF became significant when samples were induced to dielectric breakdown at a bulk sample temperature much less than a critical temperature, hypothesized to be the β‐relaxation of PMMA, i.e., *T* ≪ *T_β_* where *T_β_ =* –48°C. At bulk sample temperatures much greater than the critical temperature *T* ≫ *T_β_*, the measured amount of transferred charged at time of dielectric breakdown exponentially decreased until breakdown could no longer be initiated. Temperature dependent charge carrier mobility likely influences both the spontaneous breakdown event (sub‐microsecond timescale) and space charge retention phenomena (minutes to hours timescale) by directly influencing the amount of internal space charge density available at the time of breakdown.

The electron irradiation of bulk PMMA likely creates numerous trapping sites by enhancing the dynamic and structural disorder of the already disordered (atactic), amorphous material leading to a broadening of the DOS distribution. A potential mechanism to explain the quasi‐static charge dissipation observed at warmer temperatures may be due to increased phonon effects enabling (*i*) pathways for thermally activated hopping of charge carriers along polymer segment backbone(s) and intermolecular hopping between polymer chains, which (*ii*) decreases the probability of trap‐assisted recombination during the spontaneous DBE due to the trapping site density (i.e., space charge density) being delocalized and unavailable for ivy‐type breakdown; thus breakdown proceeds via branch‐type propagation only. Conversely, at colder temperatures quasi‐static charge dissipation appears to be restricted indefinitely; (*i*) significantly limiting pathways for thermally activating hopping along and between polymer segments, such that space charge density is sufficiently localized and available to (*ii*) enable trap‐assisted recombination during the spontaneous DBE, allowing breakdown to proceed via ivy‐type propagation, before transitioning to branch‐type propagation and then full cessation.

The proposed mechanism agrees with the carrier transport theory for amorphous materials reported in conventional, thin‐film dielectrics [43]. However, the exact origin of deep trap states in irradiated PMMA are unclear. Additionally, other types of polarization (ionic, interfacial, etc.) and their effects should be considered. On‐going experimental work is underway to validate the observations reported here with direct molecular dynamics measurements (i.e., dielectric spectroscopy and dynamic mechanical analysis). Theoretical work evaluating the efficacy of space‐charge‐limited breakdown front dynamics in bulk irradiated acrylic is currently in preparation.

## 4 Methods

Optically transparent, 5 cm cubic samples of amorphous, atactic, ultra‐high molecular weight PMMA were commercially sourced. A glass transition temperature of T_g_ ≅ 120°C was measured by differential scanning calorimetry (DSC) (TA Instruments, model DSC‐2500). A molecular weight of 1.25 Mg mol^−1^ was measured by standardized gel‐permeation chromatography (GPC) using BHT‐stabilized THF solvent against PMMA standards. Samples were placed inside a K2 Scientific freezer (model K205ULT) at –80°C for a minimum of 3 days to allow all samples to equilibrate prior to testing. Samples were then packed in dry ice and transported from the University of Maryland to an electron Dynamitron.

The cooled samples were transferred from dry ice onto a carboard‐lined surface and irradiated by a Dynamitron electron accelerator. The samples passed under the 4.5 (+3% –2%) MeV electron beam exposing one face, orthogonal to the incident beam, to an incident charge density of 10 mC m^−2^ (i.e., beam current 3 mA, beam scan width 1.5 m, scan rate of 20 cm/s). Post‐irradiation, samples were quickly repacked in dry ice and transported back to the University of Maryland, where they were returned to the control freezer (Freezer A) at –80°C.

For the first iteration (*n* = 0) of the experiment, 5 samples were transferred from the control freezer (Freezer A at *T_0_* = –80°C) and placed into an identical model variable freezer (Freezer B), set initially to *T_n = 0_* = –80°C. After another equilibration period of 3 days, 5 samples were removed from each freezer (10 samples total). The removed samples were individually placed into a custom‐built test fixture for in situ dielectric breakdown current measurements (schematic of fixture shown in Figure S5) [27]. The low‐inductance fixture was designed to maintain complete electrical contact with all sample surfaces while simultaneously initiating breakdown by driving a brass rod with a sharp tungsten tip into the center surface of the charged cube face, actuated by a pneumatic cylinder pressurized to 670 kPa. Coaxial with the brass rod was a ComPower CLCE‐1032 RF current probe used to measure the induced current produced during the breakdown event. All captured current waveforms from each of the DBEs were integrated over time for the analysis of charge transfer [28].

Once the breakdown currents for all 10 samples were measured (5 control, 5 variable), an additional 5 samples were transferred from Freezer A to Freezer B for the start of the next iteration (*n* = 1), and Freezer B was increased to its new corresponding temperature (*T_n = 1_* = –70°C). This experimental process was repeated for a total of 9 iterations (*n* = 0, 1, 2, … 8) over 27 days until the samples equilibrated in Freezer B no longer retained sufficient charge to initiate a DBE from the mechanical punch or trigger the oscilloscope when dielectric breakdown was induced inside the fixture. This criterion was met when Freezer A and Freezer B were at –80°C and 0°C, respectively. The procedural details of each iteration are shown in Table 1.

**TABLE 1:** Iteration, day count, and freezer temperature.

Iteration (*n*)	Day Count	Freezer B (variable)^a^ *T* = (*T_0_* + *n* × 10) °C
0	3	−80
1	6	−70
2	9	−60
3	12	−50
4	15	−40
5	18	−30
6	21	−20
7	24	−10
8	27	0

^a^ Freezer A (control) was held at *T_0_ = –80*°C for all days and iterations

Following the electrical measurements, optical images of the resulting electrical treeing pattern or Lichtenberg figure for each sample were taken using a Sony a7iii camera with an FE f/2.8 90 mm macro lens. Images were obtained on the face orthogonal to the incident electron beam during irradiation and on one of the side faces, which was parallel to the incident electron beam during irradiation. Samples were imaged in a dark room while illuminated from below using an LED board. White and polarized‐light images were taken of select electrical tree patterns inside the specimen bulk using a Keyence VHX 7000N Digital Microscope.

Select samples were scored circumferentially, ∼3 mm deep, using a dicing saw and then placed in liquid nitrogen for a few minutes to increase brittleness. Brittle samples were placed in a hydraulic press, positioned atop a metal fulcrum, and manually compressed until fractured. The fractured plane was inspected for exposed damage features left behind by the dielectric breakdown process. Areas of interest on the fracture surface were imaged using the z‐stack feature of a Keyence VHX 7000N Digital Microscope to obtain a single in focus image stitched together from multiple images taken over a surface roughness of ∼1 mm in z‐height.

## Author Contributions

**Kathryn M. Sturge**: data curation, methodology, investigation, writing – review and editing. **Noah Hoppis**: investigation, methodology, writing – review and editing. **Emily H. Frashure**: investigation, methodology, formal analysis, writing – review and editing. **John Cumings**: project administration, supervision, writing – review and editing, writing – original draft. **Timothy W. Koeth**: conceptualization, funding acquisition, project administration, supervision, writing – original draft, writing – review and editing. **Bryson C. Clifford**: conceptualization, data curation, formal analysis, investigation, methodology, validation, visualization, writing – review and editing, writing – original draft.

## Conflicts of Interest

The authors declare no conflicts of interest.

## Supporting information




**Supporting File 1**: advs78400‐sup‐0001‐SuppMat.docx.

**Supporting File 2**: advs78400‐sup‐0002‐SI1.png.

**Supporting File 3**: advs78400‐sup‐0003‐SI2.png.

**Supporting File 4**: advs78400‐sup‐0004‐SI3.png.

**Supporting File 5**: advs78400‐sup‐0005‐SI4.png.

**Supporting File 5**: advs78400‐sup‐0006‐SI5.png.

## Data Availability

The data are available upon reasonable request to the corresponding author.
