# Supplementary material for: Temperature Dependence of Space Charge Retention in Bulk Acrylic
Source: Adv Sci (Weinh). 2026 Oct 31:e78400. Online ahead of print. doi: 10.1002/advs.78400 (PMC13723707; doi:10.1002/advs.78400)
Supplement: Supplementary file 1 [file ADVS-9999-e78400-s005.docx]

Supporting Information

Temperature Dependence of Space Charge Retention in Bulk Acrylic

Bryson C. Clifford^*^, Kathryn M. Sturge, Noah Hoppis, Emily H. Frashure, John Cumings, and Timothy W. Koeth

^*^E-mail: bcclif02@umd.edu

Average percent change of control sample ensembles were calculated and compared to the total control sample average, which is used as the zero baseline in **Figure SI1**. Each data point or ensemble represents the average of 5 control samples measured at the control temperature of T_o_ = –80°C as a function of elapsed time in days. Average percent change of control sample ensemble measurements was found to be < 3% from baseline. The y-axis error bars represent 2σ error, in agreement with the 95% confidence interval shown in Figure 1 of the main text. A linear regression of the data points shown in Figure SI1 was used to determine potential drift of ensemble measurements taken over time, and is shown in **Table SI1**. Standard error of these measurements was found to be < 1.89.

**Figure SI1.** Average percent change of control sample ensemble measurements.

| Table SI1. Linear regression statistics of control sample ensembles. | |
| --- | --- |
| Multiple R | 0.48 |
| R Square | 0.23 |
| Adjusted R Square | 0.12 |
| Standard Error | 1.89 |
| Observations | 9 |

Average percent change of experimental sample ensembles were calculated and compared to the control sample average, which is used as the zero baseline in **Figure SI2**. Each data point or ensemble represents the average of 5 experimental samples measured at different bulk sample temperature. The y-axis error bars represent 2σ error, in agreement with the 95% confidence interval shown in Figure 1 of the main text, but are not visible due to the large scale of the y-axis. All experimental sample measurements in this temperature range decreased compared to the control sample average and thus have a negative percent change. However, the percent change values were graphed here as positive values in order to obtain an exponential fit shown (*R^2^=0.9961*).

**Figure SI2**. Average percent change of experimental sample ensemble measurements obtained in the temperature range of –40°C ≤ T ≤ 0°C.

Images of all LFs produced by space-charge-induced dielectric breakdown of control samples are shown in **Figure SI3**. Each image corresponds to one data point (diamonds) shown in the inset of Figure 1 in the main text. Each column includes 5 images which correspond to the ensemble of samples that were measured at each time interval (i.e., 5 samples were tested every 3 days from the control group). Samples were illuminated from below in a dark room to enhance contrast between the void pathways (white) and the transparent bulk material (black). The dark color intensity of the arrow (top) does not vary, indicating all samples remained at the constant control temperature of *T_o_ = –80°C* for the full duration of the experiment. The missing LFs at day 15 and 19 spontaneously induced to dielectric breakdown outside of the test fixture and were excluded from this experiment.

Images of all LFs produced by space-charge-induced dielectric breakdown of experimental samples are shown in **Figure SI4**. Each image corresponds to one data point (circles) shown in Figure 1 of the main text. Each column includes 5 images which correspond to the ensemble of samples that were measured at each temperature interval (i.e., 5 samples were tested from the experimental group at each *ΔT = +10°C* over the temperature range of *–80 ≤ T ≤ 0°C*). Samples were illuminated from below in a dark room to enhance contrast between the void pathways (white) and the transparent bulk material (black). The color shade of the arrow (bottom) lightens in proportion to increasing temperature. The missing LFs at *T = –50°C* and *T = –40°C* spontaneously induced to dielectric breakdown outside of the test fixture and were excluded from this experiment. Images that contain no LFs at *T = –10°C* correspond to samples in which space-charge-induced dielectric breakdown was not possible due to the extreme loss of space charge which occured at warmer temperatures.

**Figure SI3.** Lichtenberg figures produced from space-charge-induced dielectric breakdown of control samples.

**Figure SI4**. Lichtenberg figures obtained from space-charge-induced dielectric breakdown of experimental samples.

The following excerpt is reproduced from reference 27 with permission:

“Low Impedance Breakdown Fixture

Commercially produced 5 cm PMMA cubes have geometric tolerances of ±2 mm, requiring some means to accommodate this variation without leaving gaps around the sample. The first solution used an aluminum box to enclose the sample very loosely. A set of three aluminum pads on springs with current bonding braids to the main box were included on three adjacent inside faces of the box. These pads pushed the sample into a single corner of the box, establishing electrical contact on all sample faces. The mechanical punch passed through a plastic support bushing and current transformer (CT), which itself passed through a hole in the box top lid and topmost pad. The top of the mechanical punch was connected to the aluminum box *via* current bonding braid.

The use of bonding braids produced greater inductance than was desired. A second fixture was designed using the mechanical approach as the first (three orthogonal moving pads and three antipodal stationary pads), with reduced total impedance. A fully symmetrical system of copper finger stock was used for bonding purposes. Each moving pad was treated as a piston and surrounded by a “wear ring” of finger stock. Accordingly, the finger stock was always uniformly loaded regardless of pad position. The fixed pads were also connected to the metal frame of the fixture using a radial finger stock connection to ensure that all current paths were identical.

Drive screws were used to translate the moving pads. The anti backdrive feature of the drive screw allowed the moving pad through which the mechanical punch passed to be swapped to the opposite side of the fixture, simplifying fixture top. To reduce impedance further, the current bond braid between the mechanical punch and fixture chassis was replaced by phosphor bronze bellows. The CT was then enclosed in a metal cavity bonded to the fixture frame.”

**Figure SI5.** The second version of the low impedance current fixture used in this work. (**Left**) The fixture on its side with the sample loading door open. (**Right**) The fixture closed and upright. Current transformer cavity and bellows are visible at the top. Reproduced from [27] with permission.
